# Towards three-dimensional optical metamaterials

**DOI:** 10.1186/s40580-017-0129-7

**Published:** 2017-12-15

**Authors:** Takuo Tanaka, Atsushi Ishikawa

**Affiliations:** 1 Metamaterials Laboratory, RIKEN, 2-1 Hirosawa, Wako, Saitama 351-0198 Japan; 2 Innovative Photon Manipulation Research Team, RIKEN Center for Advanced Photonics, 2-1 Hirosawa, Wako, Saitama 351-0198 Japan; 3 Research Institute for Electronic Science, Hokkaido University, N21W10 Kita-ku, Sapporo, Hokkaido 001-0020 Japan; 4 School of Materials and Chemical Technology, Tokyo Institute of Technology, 4259 Nagatsutacho, Midoriku, Yokohama, Kanagawa 226-8503 Japan; 5 Department of Electrical and Electronic Engineering, Okayama University, 3-1-1 Tsushima-naka, Kita-ku, Okayama 700-8530 Japan

**Keywords:** Metamaterials, Plasmonics, Micro/nano-fabrication, Nanophotonics

## Abstract

Metamaterials have opened up the possibility of unprecedented and fascinating concepts and applications in optics and photonics. Examples include negative refraction, perfect lenses, cloaking, perfect absorbers, and so on. Since these metamaterials are man-made materials composed of sub-wavelength structures, their development strongly depends on the advancement of micro- and nano-fabrication technologies. In particular, the realization of three-dimensional metamaterials is one of the big challenges in this research field. In this review, we describe recent progress in the fabrication technologies for three-dimensional metamaterials, as well as proposed applications.

## Introduction

Metamaterials are man-made materials composed of metallodielectric structures [1]. Since their unit elements are designed to be smaller than the wavelength of light, these metamaterials work as quasi-homogeneous materials; this is the reason why they are referred to as meta-“materials” not meta-“structures”. The most important aim of engineering such materials is to obtain complete control of light waves using the unprecedented optical properties and functionalities that arise from these artificial structures.

A perfect lens achieved by negative index materials is one of such unprecedented functionalities [2]. The refractive index of a material is defined by the product of the square root of the relative permittivity and the square root of the relative magnetic permeability. Since all natural materials lose their magnetic responses and its permeability becomes unity in the optical frequency region, negative index materials are never found in nature. Recent achievements in the creation of optical magnetism by using metamaterials have opened up the possibility of novel optical phenomena and effects that were previously considered to be unrealizable [3]. In addition to negative index materials, a wide variety of potential applications have been predicted and demonstrated in the past decade, such as invisible cloaking, designer dispersion, absorption management, and so on [4].

One of the trends in this research field is increasing the working frequency of metamaterials, which began in the microwave frequency regime, up to the visible light frequency regions, which is several hundreds THz [5]. Many efforts have been made by researchers in theoretical studies for realizing optical metamaterials, and now we know that what kinds of materials and resonant structures are appropriate for realizing optical metamaterials. The recent advances in micro- and nano-fabrication technologies have brought such ideas to reality, having a huge impact in a wide range of optics and photonics research [6]. However, since current fabrication technologies, such as photolithography and electron-beam (EB) lithography, are based on two-dimensional (2D) patterning techniques, the realization of true three-dimensional (3D) optical metamaterials is still a challenge [7]. In this review, we focus on recent progresses made in the fabrication techniques for 3D metamaterial structures [8].

## 3D metamaterials vs. 3D functional metamaterials

The term “3D metamaterials” is usually used to represent “bulk metamaterials” or “stereoscopic metamaterials”. However, there are actually two different meanings of the word “3D”. One is that the shape of the metamaterial itself is 3D and not planar. The other meaning comes from the degrees of freedom of the functionality of the metamaterial. In the first section of this review, we would like to emphasize this difference; that is the difference between the dimensions of the metamaterial’s shape and that of its functionality.

**Fig. 1:**
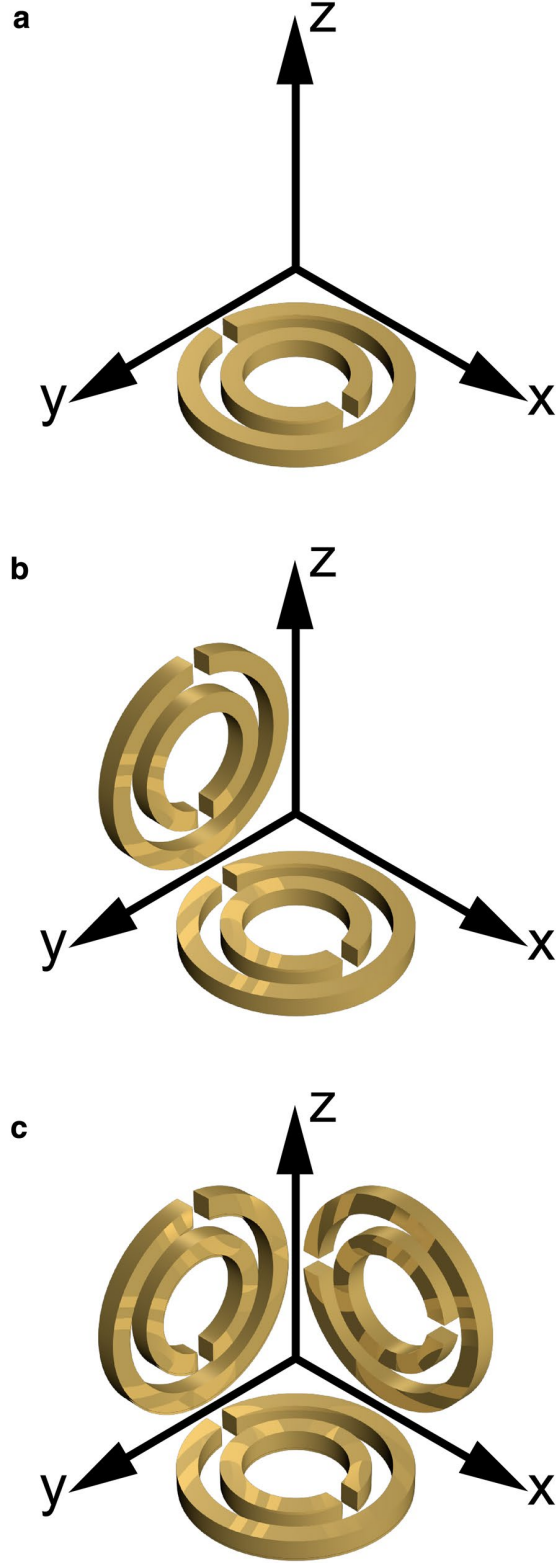
3D metamaterials vs. 3D functional metamaterials. **a** 2D structure but functionality of metamaterial is 1D. **b** 3D structure but 2D funtional metamaterials, and **c** 3D in both structure and functionality metamterial

Optical metamaterials use resonant structure as their building blocks (unit elements). One of the most well-known unit elements is the “split-ring resonator (SRR)”, which was proposed by Pendry et al. [9]. An SRR consists of metal rings with several slits; the metal rings work as magnetic antenna and inductors and the slits introduced in the rings work as capacitors. The operation of the SRR is based on an LC resonant circuit. Since there is no antenna structure that has isotropic radiation property, metamaterials made with SRR arrays also inevitably become anisotropic. Figure 1a shows an SRR placed on the x–y plane. This SRR can couple with a magnetic field that oscillates in the z-direction, and it does not interact with a field that oscillates in the x–y plane. Therefore, from the viewpoint of the degree of freedom of the magnetic interactions, this unit structure is categorized as a one-dimensional (1D) metamaterial element. When these structures are integrated into a 2D array, the structure is defined as a 2D structure. However, the metamaterial functionality is still 1D. Moreover, when such 2D structures are stacked in the z-direction, they form a bulk structure, but the metamaterial functionality consistently remains 1D.

In this sense, we recognize that the dimension of the functionality of a metamaterial is different from the dimension of its shape. To increase the degree of freedom of metamaterial functions, additional different orientated structures are necessary, as shown in Fig. 1b, c. Here, when we put an additional SRR in the x–z plane, the metamaterial exhibits 2D optical functions, and adding an SRR in the y–z plane, the metamaterial gains complete 3D controllability of light waves. In this paper, we define the terms “bulk metamaterials” and “3D metamaterials” as metamaterials whose structure is distributed in x–y–z space irrespective of the dimension of its functionality, and we use the term “3D-functional metamaterials” for those that have complete 3D functionality.

## Stacking of planar structures in three dimensions

Stacking 2D planar layers is the easiest way to fabricate a bulk structure. The recent progress in micro- and nano-fabrication techniques, such as photolithography and EB lithography, enable us to fabricate 2D structures on the surface of a solid substrate with nanometer-scale accuracy.

**Fig. 2:**
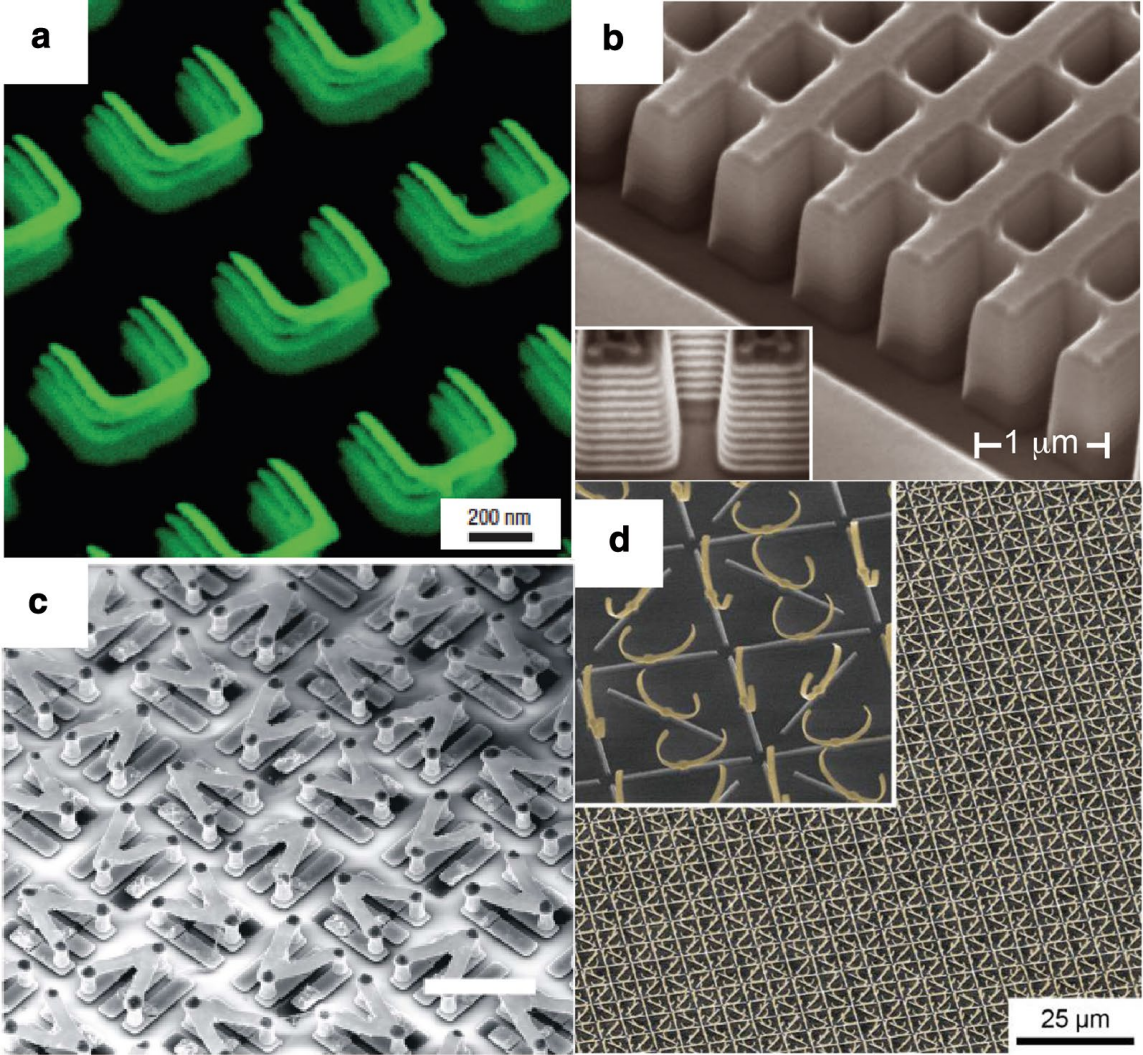
From stacked 2D to 3D metamaterials. **a** Magnetic metamaterial made of four-layer SRR structure [10]. **b** Negative-refractive-index metamaterial made of 21-layer fishnet structure [17]. **c** Chiral switching metamaterial made of 3D metallic structure [25], and **d** isotropic metamaterial made of 3D SRR structure [82]

In 2008, Liu et al. reported a photonic metamaterials in which four SRR array structures are stacked by using a layer-by-layer technique [10]. In their experiment, they fabricated 430 nm by 380 nm square SRRs with an 80 nm linewidth using EB lithography, and then stacked four layers one-by-one with 70 nm-thick polymer spacing layers between them. Figure 2a shows an oblique-incidence view taken by a scanning electron microscope (SEM). In 2009, the same technique was applied to make stereo-SRR dimer metamaterials with various twist angles [11]. The same team used this technique to create a plasmonic induced transparency (PIT) in a multi-layered metamaterial in which gold nano-rods are stacked on an underlying gold nano-wire pair [12]. Based on dipole–quadrupole coupling in this stacked PIT structure, they observed a PIT band in the absorption dips of the upper Au nano-rods. Multi-layer stacking of SRRs also provides huge optical activity [13], and this was also applied to plasmonic rulers to determine the nanoscale structures of molecules such as proteins, DNA, and so on [14].

In 2012, Kante et al. proposed a two-layer closed nano-rings structure fabricated by the multiple EB lithography method [15]. In this technique, two layers of closed gold nano-ring structures are fabricated on a quartz substrate with an SU-8 photoresist spacing layer. Alignment of the two rings is precisely controlled by the EB lithography technique. When one of the rings is fully shifted with respect to the other ring, due to the spectral overlap of symmetric and asymmetric modes and Fano-type interference between them, a negative index band appears in the 1.9 µm wavelength range.

About the realization of negative index metamaterials, Garcia-Meca et al. fabricated a multi-layered fishnet metamaterial using alternate deposition of silver layers and hydrogen silsesquioxane-based resist and a focused ion-beam milling technique [16]. In 2008, negative refraction was experimentally demonstrated in the near-infrared regime (1200–1700 nm wavelength) by Valentine et al. [17]. A 21-layer fishnet structure consisting of alternating layers of 30 nm silver and 50 nm magnesium fluoride was patterned by focused ion-beam (FIB) milling, as shown in Fig. 2b. Additional FIB milling was applied to the multi-layer fishnet structure to form a prism shape, and the effective refractive index was experimentally evaluated by measuring the absolute angle of refraction. Xu et al. used a multi-layer structure of Ag and TiO_2_ to fabricate a negative index metamaterial in the ultraviolet region and demonstrated a flat lens effect [18]. Silicon rods separated by a silicon dioxide layer enabled the realization of zero-index metamaterials in the near-infrared region (λ = 1.4 µm) [19].

In 2011, Chanda et al. reported a flexible 3D optical negative index metamaterial fabricated by a nanotransfer printing technique. This technique is based on a nanoimprint lithography method. A multilayer structure was deposited on a soft imprinting mold by means of EB evaporation, and this patterned multilayer structure was transferred to the target substrate by contacting the mold against the substrate. They used a PDMS film for the substrate and demonstrated flexible 3D metamaterials with a negative index of refraction in the near infrared regime [20].

Double-exposure EB lithography was applied to make a U-shaped upright 3D metamaterial structure [21]. A U-shaped upright SRR resonantly interacts with the magnetic components of incident light waves, and field enhancements of 16-times and 4-times were demonstrated at the center and two prongs of the SRR, respectively.

Chirality requires a 3D structure and never occurs in 2D ones. Left-handed and right-handed twisted double-layer cross structures were fabricated, and the polarization dependences on left- and right-handed circularly polarized incident light were compared [22].

Multilayer photolithography and electroplating techniques were utilized to fabricate 3D self-standing SRR resonator arrays on a silicon substrate [23]. Since the fabricated SRR arrays are real 3D structures but they are aligned in one direction, the fabricated metamaterial structure has only one degree of freedom of the magnetic interaction. Zhang et al. used the multilayer photolithography and lift-off techniques to fabricate a THz metamaterial structure that consists of 3D unit cells. In 2009, they applied this technique to demonstrate a chiral negative index of refraction in the 1 THz region [24]. In 2012, they also applied the same fabrication technique to make chiral switchable metamaterials that are controlled by photoexcitation with a femtosecond near infrared laser, as shown in Fig. 2c [25]. Because these results use photolithogprahy technique, the size of the structures are limited down to several hundreds nanometers.

## Two-photon absorption techniques

Photolithography and EB lithography have already been widely used as basic tools for the fabrication of 2D metamaterials, and these techniques can be extended to the fabrication of 3D metamaterials by stacking. Although these techniques demonstrate good productivity in 2D fabrication, the fabrication of 3D structures with 3D functions has never been achieved. On the other hand, micro-stereolithography [26, 27] and FIB chemical vapor deposition (FIB-CVD) [28] enable the creation of arbitrary 3D structures. However, these techniques are still based on layer-by-layer fabrication and do not inherently have 3D spatial resolution.

The process of two-photon absorption (TPA) was first predicted by Göppert-Mayer in 1931 [29]. In contrast to the one-photon absorption (OPA), the absorption probability in TPA is proportional to the square of the light intensity, and thus, light absorption is localized inside the focal point. At the same time, successive chemical or physical reactions are confined in a small volume with spatial resolution in three dimensions. As a result, unlike other fabrication techniques, the TPA process inherently has 3D resolution, with potential applications in fluorescence microscopy [30], optical data storage [31], and lithographic fabrication [32, 33]. On the other hand, due to the diffraction limit, the practical spatial resolution of these techniques are almost limited down to several hundreds nanometers, while they have nonlinear properties to the intensity distribution of the laser spots. In this section, several 3D fabrication techniques based on TPA are reviewed.

### Two-photon photopolymerization for 3D polymer structures

**Fig. 3:**
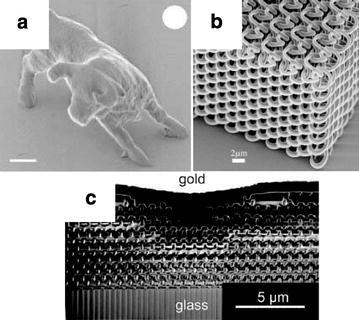
Fabrication techniques for 3D polymer structures. Two-photon polymerization technique for **a** 3D nano-structures [35] and **b** 3D photonic crystal [38]. **c** 3D carpet cloaking device fabricated by two-photon polymerization [39]

A fabrication technique based on a combination of micro-stereolithography and TPA was first demonstrated by Maruo et al. in 1997 and offers great potential for the production of 3D polymeric micro/nano structures [34]. A near-infrared femtosecond pulsed laser beam is tightly focused in a photo-polymerizable resin with a high-NA objective lens. Because in the TPA process, the solidification of the resin occurs only at the focal point, where a large number of photons simultaneously exist, by scanning the laser beam spot three-dimensionally inside the resin, 3D polymeric structures with arbitrary shapes can be fabricated. The nonlinear nature of the TPA process offers both 3D spatial resolution and the ability to achieve sub-diffraction-limit fabrication, as shown in Fig. 3a [35].

With such a 3D laser fabrication capability, so-called “direct laser writing” (DLW), a wide variety of 3D optical functional devices can be realized. Photonic crystals (PCs), which consist of periodic dielectric structures with dimensions on the wavelength scale, are one of the most important applications for optical communications, but the realization of full 3D photonic bandgap (PBG) crystals is still a challenge, even when employing cutting-edge semiconductor processes [36]. By using DLW, Deubel et al. demonstrated the first fabrication of a large-scale face-centered-cubic (f.c.c.) PC in 2004 [37]. They used a commercially available photoresist, SU-8, in the DLW process and fabricated a 3D PC that exhibits PBGs at near-infrared wavelengths from 1.3 to 1.7 µm. The DLW technique allows even complex 3D photonic structures that cannot be fabricated in a layer-by-layer built-up manner. Seet et al. demonstrated the fabrication of 3D spiral-architecture PCs using SU-8 photoresist, as shown in Fig. 3b, and observed PBGs at infrared wavelengths [38]. Based on 3D PCs fabricated by the DLW technique, Ergin et al. recently realized a 3D photonic metamaterial, shown in Fig. 3c, that exhibits so-called invisibility cloaking for unpolarized infrared light [39]. They designed and fabricated a 3D f.c.c. wood-pile PC with a tailored filling fraction on a Au mirror surface and demonstrated carpet cloaking at near-infrared wavelengths [40]. The spatial resolution of the DLW technique is naturally limited by the nonlinear characteristics of the TPA process and is typically as low as 120 nm for an excitation wavelength of 780 nm [35]. These limitations were recently improved by introducing a novel microscopy technique based on stimulated-emission-depletion (STED) [41, 42]. Fischer et al. recently demonstrated STED-DLW by using a specially synthesized photoresist system and achieved a spatial resolution as low as 65 nm for an excitation (depletion) wavelength of 810 (532) nm [43, 44].

### Two-photon processes for 3D metal structures

Although two-photon-induced photopolymerization offers wide versatility in the fabrication of 3D complex micro/nanostructures, only dielectric photonic structures have been developed so far. To gain more functionality in photonic and plasmonic applications, the development of 3D metallodielectric structures is important and highly desired. There are several reports on the fabrication of 3D metallodielectric structures based on the TPA process, and they can be categorized into two techniques: (i) electroless plating of a polymer template [45] and (ii) photoreduction of metal ions in a polymer matrix/solution [33, 46].

**Fig. 4:**
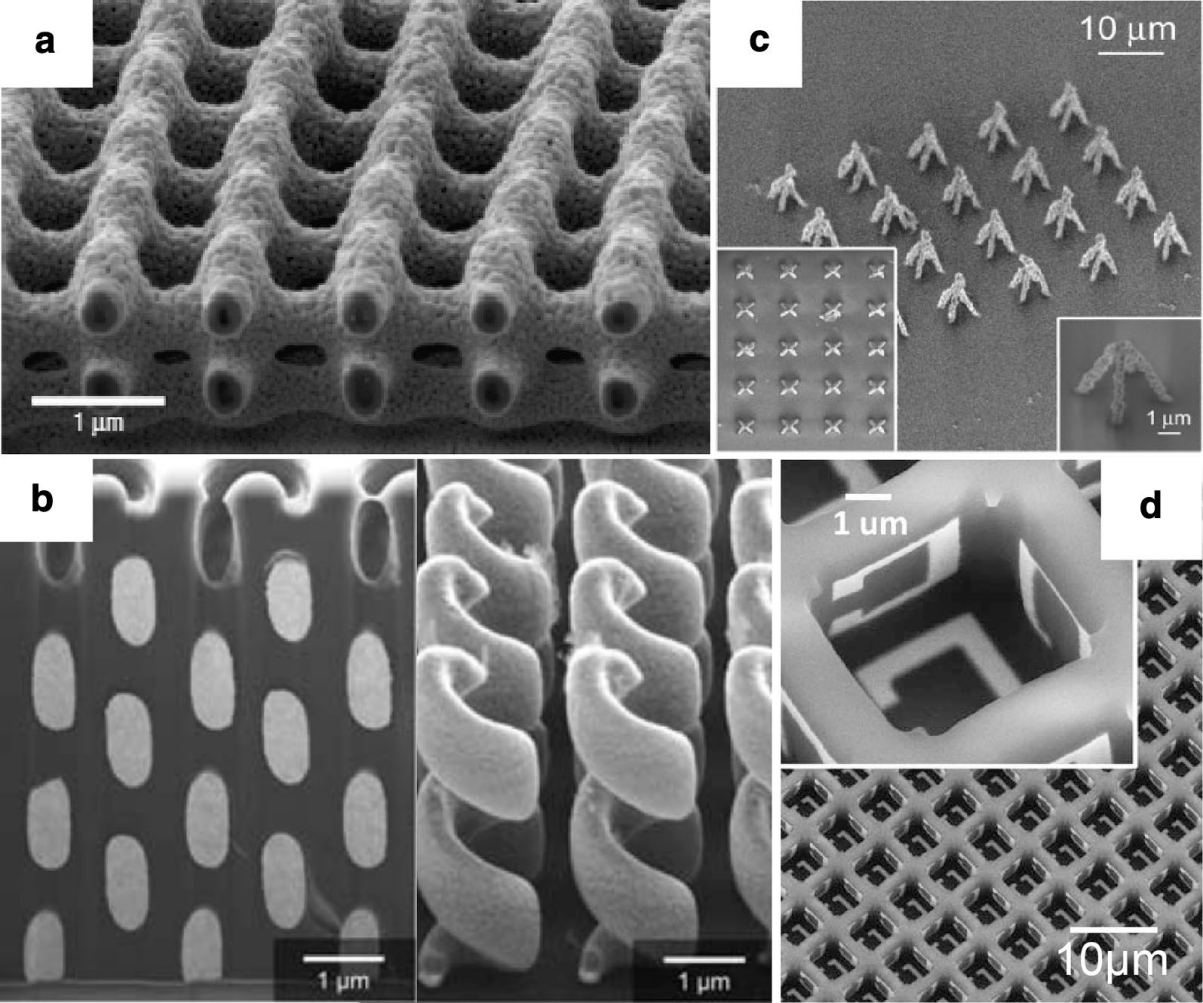
Fabrication techniques for 3D metallic structures. **a** Silver chemical vapor deposition onto polymer template [50]. **b** Electroless plating in SU-8 3D template [51]. **c** Two-photon-induced photoreduction of metal ions [61], and **d** projection lithography technique [65]

Electroless plating of 3D polymer structures was first reported by Farrer et al., and they demonstrated gold/copper coating of selectively functionalized acrylic/methacrylic structures [45]. Formanek et al. demonstrated selective silver coating of 3D polymer structures by chemically modifying the polymer surface with stannous chloride (SnCl_2_). They also employed a microlens array to realize parallel fabrication and demonstrated mass-production of 3D metallodielectric microstructures over a large sample area [47, 48]. Takeyasu et al. also demonstrated a similar selective coating technique by directly mixing methacrylamide in a resin to selectively activate/non-activate the polymer surface [49]. Rill et al. fabricated 3D metallodielectric microstructures by combining the DLW process and atomic-layer deposition (ALD), as shown in Fig. 4a. In their process, an SU-8 template of a 3D structure was first coated with SiO_2_ using ALD, followed by a CVD process to realize 3D deposition onto the polymer surface [50]. In contrast to such surface coating techniques, Gansel et al. fabricated 3D metallodielectric microstructures by employing the electrochemical deposition of gold onto an exposed positive-tone photoresist. After removing the polymer template by plasma etching, they demonstrated gold-helix structures, shown in Fig. 4b, that work as a compact broadband circular polarizer [51]. These techniques have been extensively studied recently and applied to the development of a variety of functional optical/mechanical 3D devices [52–56].

Photoreduction of metal ions in polymer matrix was first reported by Wu et al., and they demonstrated the fabrication of silver microstructures inside a SiO_2_ sol–gel matrix based on image formation and developing processes [44]. Similar techniques were also reported by Duan et al. using titanium ions for the fabrication of functional composite materials [57, 58]. On the other hand, Stellacci et al. demonstrated improved reduction properties in a polymer matrix by chemically modifying metal nanoparticles [59]. They realized electrically conductive silver or gold 3D structures in a polymer matrix, but the resistivity of the metal structures is still small compared with that of bulk metal, suggesting that the fabricated structures were not fully connected. To demonstrate fully connected 3D metallic structures, Tanaka et al. proposed the two-photon-induced reduction of metal ions in aqueous solution and demonstrated 3D metallic structures with superior electrical conductivity [46]. The reduction property and spatial resolution could be improved further by introducing a two-photon sensitive dye for high-efficiency photoreduction [60] and surfactant molecules to avoid unwanted crystal growth, as shown in Fig. 4c [61]. These techniques have recently been applied to the development of magnetic metamaterials at infrared frequencies [62].

### Other techniques for 3D metallodielectric structures

**Fig. 5:**
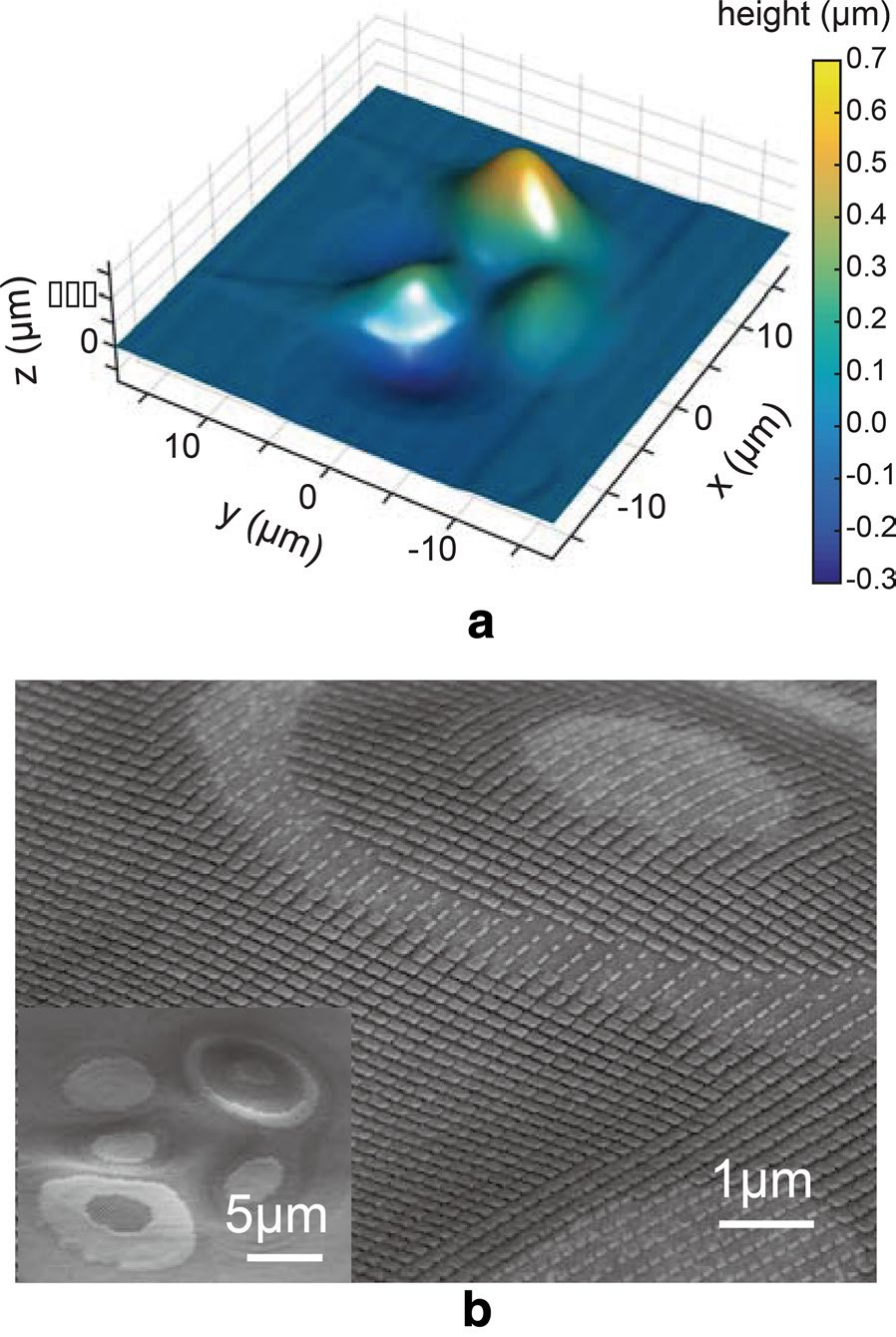
Metasurface structure for carpet cloaking of 3D objects [72]. **a** An AFM image of a 3D object with multiple bumps and dents. **b** SEM image of an object onto which a metasurface skin cloak has been fabricated. The inset shows an enlarged image of the entire object

Grayscale photolithography has been widely applied to fabricate 3D polymer structures, and the recent development of digital light processing has enhanced its capability for even complex 3D fabrication [63, 64]. However, the fabricated structures are still limited to surface profiles on photoresist, and only 3D surface modifications have been achieved. To extend the 2D capability of conventional photolithography to three-dimensions, Burckel et al. proposed membrane projection lithography, as shown in Fig. 4d [65, 66]. Although this technique is still based on the conventional planar lithography, one can create out-of-plane metallic structures, enabling the realization of 3D metamaterials. A 2D counterpart of a metamaterial, i.e., a metasurface, has been proposed as a simple yet powerful concept to mold the flow of light in a desired manner [67, 68]. Although most of the metasurfaces demonstrated so far are based on 2D planar structures, extending the technique into the third dimension is the next step to gain more functionality for versatile photonic applications [69–71]. Recently, Ni et al. demonstrated a metasurface on a 3D arbitrarily shaped object to realize carpet cloaking at visible wavelengths, as shown in Fig. 5 [72]. The height information of the 3D object was first obtained by using an atomic force microscope (AFM), and then metasurface 2D structures were patterned at each local position by using a standard EB lithography technique with precise focus alignment.

## Self-organization and templating techniques for large-area metamaterials

The fabrication techniques that are referred to as “top-down techniques”, such as photolithography, EB lithography, and DLW, have the advantage that they can fabricate diverse patterns with precise controllability of the pattern alignment. However, these techniques are intrinsically time- and energy-consuming, and it is still challenging to fabricate a large amount of micro-/nanostructures. On the other hand, bottom-up approaches based on self-organized fine structures of metamaterial templates are effective tools for the mass-production of metamaterial structures with nanometer scale or, sometimes, molecular scale feature sizes.

In addition, as written in below, the self-organization techniques have a potential to fabricate 3D structures with 3D metamaterials functions because some of them can intrinsically create randomly dispersed/oriented metamaterial unit elements.

Yao et al. used a nano-porous alumina produced by electrochemical anodization to make a silver nanowire array [73]. Silver nanowire arrays of 60 nm in diameter, 110 nm center-to-center distance, and 4.5 or 11 µm in length were fabricated by electrochemical deposition of silver into the pores. Negative refraction was experimentally observed at wavelengths of 660 and 780 nm.

According to theoretical investigations of optical metamaterial structures, metal ring structures with gaps of several nanometers work as the resonant units of metamaterials [74]. To form such structures, a DNA-templating method was proposed by Watanabe et al. [75, 76]. A chemically synthesized gold nanoparticle was bound to a single strand of DNA with thiol moiety. When three DNA strands with Au nanoparticles are hybridized with each other, a triangular DNA template structure is automatically formed, and then three Au nanoparticles are assembled into a trimer ring structure with nanometer scale gaps. Yang et al. proposed thermodynamically feedback-driven self-assembling technique to symmetry-breaking gold nanorod dimers using DNA [77]. Kuzyk et al. developed DNA-based self-assembling method of chiral plasmonic structures and demonstrated circular dichroism effect in the visible light region [78].

Self-organization of colloidal particles on a substrate can be used for 3D nanofabrication templates. Fredriksson et al. used the hole–mask colloidal lithography technique [79]. In the first step, randomly dispersed particles are used as a mask for metal evaporation to make a metal hole–mask pattern. Using the metal hole–mask and plasma etching, a PMMA sacrificial film was patterned; then, materials were evaporated on the substrate through the PMMA pattern, and various patterns such as nanodisks, oriented elliptical nanostructures, nano-cone arrays, nanodisk pairs, and so on were fabricated. Lodewijks and his colleagues also used self-assembled nanospheres as a mask to make large-area double fishnet metamaterial structures [80]. Aoki et al. demonstrated a magnetic self-assembly technique to form resonant unit cells of metamaterials [81]. Randomly dispersed gold core–shell micro-particles in water are attracted to the equator plane of the center polystyrene bead and automatically formed a necklace resonator structure by the application of an external magnetic field.

Nastaushev et al. demonstrated the self-organized creation of micro- and nano-tubes from strained metal bifilms [82]. They examined which combination of materials and Au/Ti bifilm has the best properties for the tube formation. This structure also works as a so-called Swiss-roll resonant element. Mei et al. used the same technique to form an integrated microtube array, and they examined the photoluminescence spectrum of SiO/SiO_2_ microtubes [83].

Chen et al. have developed a stress-driven assembly method for self-standing three-dimensional split-ring resonators using intrinsic stresses in thin metal films [84]. They demonstrated a 3D SRR array made of Al. In 2015, they applied the technique to realize three-dimensional isotropic metamaterials [85]. The materials for SRR were changed from Al to Au/Ni bi-films. The fabrication process and a scanning electron micrograph of the 3D SRR array are already shown in Fig. 2d. By using the fabricated 3D metamaterials, they demonstrated isotropic optical responses in the 30 THz region, and an extremely low refractive index of 0.35, which is lower than the vacuum refractive index, was also demonstrated in this frequency region.

## Conclusion and outlook

The study of metamaterials is still young, but owing to the rapid development of micro- and nano-fabrication technologies, three-dimensional optical metamaterials are no longer mere conceptual ideas but have become a practical reality. However, the total area or volume of the three-dimensional metamaterials are still small, and when we are thinking about the practical applications of metamaterials, it is crucial to develop new mass-producible fabrication techniques for nano-scale 3D metallodielectric structures.

## References

[1] Smith DR, Pendry JB, Wiltshire MCK (2004). Science.

[2] Pendry JB (2000). Phys. Rev. Lett..

[3] Shalaev VM (2007). Nat. Photon..

[4] Zheludev NI (2010). Science.

[5] Soukoulis CM, Linden S, Wegener M (2007). Science.

[6] Ishikawa A, Tanaka T, Kawata S (2005). Phys. Rev. Lett..

[7] Soukoulis CM, Wegener M (2011). Nat. Photon..

[8] Ishikawa A, Tanaka T (2013). IEEE J. Select. Topics Quantum Electron..

[9] Pendry JB, Holden AJ, Robbins DJ, Stewart WJ (1999). IEEE Trans. Microw. Theory Tech..

[10] Liu N, Guo H, Fu L, Kaiser S, Schweizer H, Giessen H (2008). Nat. Mater..

[11] Liu N, Liu H, Zhu S, Giessen H (2009). Nat. Photon..

[12] Liu N, Langguth L, Weiss T, Kästel J, Fleischhauer M, Pfau T, Giessen H (2009). Nat. Mater..

[13] Decker M, Zhao R, Soukoulis CM, Linden S, Wegener M (2010). Opt. Lett..

[14] Liu N, Hentschel M, Weiss T, Alivisatos AP, Giessen H (2011). Science.

[15] Kante B, Park Y-S, O’Brien K, Shuldman D, Lanzillotti-Kimura ND, Wong ZJ, Yin X, Zhang X (2012). Nat. Commun..

[16] García-Meca C, Hurtado J, Martí J, Martínez A (2011). Phys. Rev. Lett..

[17] Valentine J, Zhang S, Zentgraf T, Ulin-Avila E, Genov DA, Bartal G, Zhang X (2008). Nature.

[18] Xu T, Agrawal A, Abashin M, Chau KJ, Lezec HJ (2013). Nature.

[19] Moitra P, Yang Y, Anderson Z, Kravchenko II, Briggs DP, Valentine J (2013). Nat. Photon..

[20] Chanda D, Shigeta K, Gupta S, Cain T, Carlson A, Mihi A, Baca AJ, Bogart GR, Braun P, Rogers JA (2011). Nat. Nanotechnol..

[21] Chen WT, Chen CJ, Wu PC, Sun S, Zhou L, Guo G-Y, Hsiao CT, Yang K-Y, Zheludev NI, Tsai DP (2011). Opt. Express.

[22] Decker M, Ruther M, Kriegler CE, Zhou J, Soukoulis CM, Linden S, Wegener M (2009). Opt. Lett..

[23] Fan K, Strikwerda AC, Tao H, Zhang X, Averitt RD (2011). Opt. Express.

[24] Zhang S, Park Y-S, Li J, Lu X, Zhang W, Zhang X (2009). Phys. Rev. Lett..

[25] Zhang S, Zhou J, Park Y-S, Rho J, Singh R, Nam S, Azad AK, Chen H-T, Yin X, Taylor AJ, Zhang X (2012). Nat. Commun..

[26] Zhang X, Jiang XN, Sun C (1999). Sens. Actuators.

[27] Zhou F, Bao Y, Cao W, Stuart CT, Gu J, Zhang W, Sun C (2011). Sci. Rep..

[28] Matsui S, Kaito T, Fujita J, Komuro M, Kanda K, Haruyama Y (2000). J. Vaccum Sci. Technol. B.

[29] Göppert-Mayer M (1931). Ann. Phys..

[30] Denk W, Strickler JH, Webb WW (1990). Science.

[31] Parthenopoulos DA, Rentzepis PM (1989). Science.

[32] Zhou W, Kuebler SM, Braun KL, Yu T, Cammack JK, Ober CK, Perry JW, Marder SR (2002). Science.

[33] Wu P-W, Cheng W, Martini IB, Dunn B, Schwartz BJ, Yablonovitch E (2000). Adv. Mater..

[34] Maruo S, Nakamura O, Kawata S (1997). Opt. Lett..

[35] Kawata S, Sun H-B, Tanaka T, Takada K (2001). Nature.

[36] Noda S, Tomoda K, Yamamoto N, Chutinan A (2009). Science.

[37] Deubel M, Freymann GV, Wegener M, Pereira S, Busch K, Soukoulis CM (2004). Nat. Mater..

[38] Seet KK, Mizeikis V, Matsuo S, Juodkazis S, Misawa H (2005). Adv. Mater..

[39] Ergin T, Stenger N, Brenner P, Pendry JB, Wegener M (2010). Science.

[40] Li J, Pendry JB (2008). Phys. Rev. Lett..

[41] Hell SW, Wichmann J (1994). Opt. Lett..

[42] Hell SW (2007). Science.

[43] Fischer J, von Freymann G, Wegener M (2010). Adv. Mater..

[44] Fischer J, Wegener M (2011). Adv. Opt. Express.

[45] Farrer RA, LaFratta CN, Li L, Praino J, Naughton MJ, Saleh BEA, Teich MC, Fourkas JT (2006). J. Am. Chem. Soc..

[46] Tanaka T, Ishikawa A, Kawata S (2006). Appl. Phys. Lett..

[47] Formanek F, Takeyasu N, Tanaka T, Chiyoda K, Ishikawa A, Kawata S (2006). Appl. Phys. Lett..

[48] Formanek F, Takeyasu N, Tanaka T, Chiyoda K, Ishikawa A, Kawata S (2006). Opt. Express.

[49] Takeyasu N, Tanaka T, Kawata S (2008). Appl. Phys. A.

[50] Rill MS, Plet C, Thiel M, Staude I, von Freymann G, Linden S, Wegener M (2008). Nat. Mater..

[51] Gansel JK, Thiel M, Rill MS, Decker M, Bade K, Saile V, von Freymann G, Linden S, Wegener M (2009). Science.

[52] Radke A, Gissibl T, Klotzbücher T, Braun PV, Giessen H (2011). Adv. Mater..

[53] Schaedler TA, Jacobsen AJ, Torrents A, Sorensen AE, Lian J, Greer JR, Valdevit L, Carter WB (2011). Science.

[54] Zheng X, Lee H, Weisgraber TH, Shusteff M, DeOtte J, Duoss EB, Kuntz JD, Biener MM, Ge Q, Jackson JA, Kucheyev SO, Fang NX, Spadaccini CM (2014). Science.

[55] Meza LR, Das S, Greer JR (2014). Science.

[56] Kaschke J, Wegener M (2015). Opt. Lett..

[57] Duan X-M, Sun H-B, Kaneko K, Kawata S (2004). Thin Solid Films.

[58] Fukushima M, Yanagi H, Hayashi S, Sun H-B, Kawata S (2004). Physica E.

[59] Stellacci F, Bauer CA, Mayer-Friedrichsen T, Wenseleers W, Alain V, Kuebler SM, Pond SJK, Zhang Y, Marder SR, Perry JW (2002). Adv. Mater..

[60] Ishikawa A, Tanaka T, Kawata S (2006). Appl. Phys. Lett..

[61] Cao Y-Y, Takeyasu N, Tanaka T, Duan X-M, Kawata S (2009). Small.

[62] Ishikawa A, Tanaka T, Kawata S (2007). Appl. Phys. Lett..

[63] Totsu K, Esashi M (2005). J. Vaccum Sci. Technol. B.

[64] Rammohan A, Dwivedi PK, Martinez-Duarte R, Katepalli H, Madou MJ, Sharma A (2011). Sens. Actuators B Chem..

[65] Burckel DB, Wendt JR, Eyck GAT, Ginn JC, Ellis AR, Brener I, Sinclair MB (2010). Adv. Mater..

[66] Burckel DB, Wendt JR, Eyck GAT, Ellis AR, Brener I, Sinclair MB (2010). Adv. Mater..

[67] Yu N, Genevet P, Kats MA, Aieta F, Tetienne J-P, Capasso F, Gaburro Z (2011). Science.

[68] Yu N, Capasso F (2014). Nat. Mater..

[69] Ni X, Kildishev AV, Shalaev VM (2013). Nat. Commun..

[70] Huang L, Chen X, Mühlenbernd H, Zhang H, Chen S, Bai B, Tan Q, Jin G, Cheah K-W, Qiu C-W, Li J, Zentgraf T, Zhang S (2013). Nat. Commun..

[71] Yin X, Ye Z, Rho J, Wang Y, Zhang X (2013). Science.

[72] Ni X, Wong ZJ, Mrejen M, Wang Y, Zhang X (2015). Science.

[73] Yao J, Liu Z, Liu Y, Wang Y, Sun C, Bartal G, Stacy AM, Zhang X (2008). Science.

[74] Ishikawa A, Tanaka T, Kawata S (2007). J. Opt. Soc. B.

[75] Ohshiro T, Zako T, Watanabe-Tamaki R, Tanaka T, Maeda M (2010). Chem. Commun..

[76] Watanabe-Tamaki R, Ishikawa A, Tanaka T, Zako T, Maeda M (2012). J. Phys. Chem. C.

[77] Yang S, Ni X, Yin X, Kante B, Zhang P, Zhu J, Wang Y, Zhang X (2014). Nat. Nanotechnol..

[78] Kuzyk A, Schreiber R, Fan Z, Pardatscher G, Roller EM, Högele A, Simmel FC, Govorov AO, Liedl T (2012). Nature.

[79] Fredriksson H, Alaverdyan Y, Dmitriev A, Langhammer C, Sutherland DS, Zach M, Kasemo B (2007). Adv. Mater..

[80] Lodewijks K, Verellen N, Roy WV, Moshchalkov V, Borghs G, Dorpe PV (2011). Appl. Phys. Lett..

[81] Aoki K, Furusawa K, Tanaka T (2012). Appl. Phys. Lett..

[82] Nastaushev YV, Prinz VY, Svitasheva SN (2005). Nanotechnology.

[83] Mei Y, Huang G, Solovev AA, Urena EB, Monch I, Ding F, Reindl T, Fu RKY, Chu PK, Schmidt OG (2008). Adv. Mater..

[84] Chen CC, Hsiao CT, Sun S, Yang K-Y, Wu PC, Chen WT, Tang YH, Chau Y-F, Plum E, Guo G-Y, Zheludev NI, Tsai DP (2012). Opt. Express.

[85] Chen CC, Ishikawa A, Tang Y-H, Shiao M-H, Tsai DP, Tanaka T (2015). Adv. Opt. Mater..

